# Efficacy and prognostic factors for successful treatment of port-wine stains by 577-nm yellow laser: a cohort study on 42 patients

**DOI:** 10.1007/s10103-025-04350-w

**Published:** 2025-02-14

**Authors:** Essamelden M. Mohamed, Hazem L. Abdel-Aleem, Mofreh Mansour, Mahmoud A. Rageh

**Affiliations:** 1 Department of Dermatology, Faculty of Medicine, Al-Azhar University, Assiut, Egypt; 2 Department of Dermatology, Faculty of Medicine for Girls, Al-Azhar University, Damietta, Egypt; 3 Department of Dermatology, Faculty of Medicine, Al-Azhar University, Cairo, Egypt

**Keywords:** Port-wine stain, Birth marks, Vascular laser, Yellow laser

## Abstract

A port-wine stain (PWS) is a congenital capillary abnormality with an incidence of 0.3–0.5%. Although several other types of lasers have been used to treat PWSs, few studies have focused on the factors that affect the outcome of 577-nm yellow lasers. We aimed to assess the efficacy and prognostic factors affecting the PWS treatment by 577-nm yellow laser. This study was carried out on 42 patients with PWS. Each patient received 6–10 treatment sessions with a 577-nm yellow laser at 4-week intervals. Treatment efficacy was considered adequate when improvement of ≥ 80% of the lesion occurred. After treatment, marked improvement occurred in 7 (16.7%) patients, moderate improvement in 17 (40.5%) patients, mild improvement in 10 (23.8%) and 8 (19%) patients showed poor improvement. There was a significant association between improvement and gender of the patients and site of lesion (*p* = 0.028, *p* = 0.001, respectively). However, linear regression analysis showed that the site of the lesion can significantly predict the improvement (*p* < 0.001), while other baseline characteristics were not associated and cannot act as predictors for improvement. Yellow laser is a successful therapy choice for PWS, with a statistically significant improvement and minimal adverse effects. No significant association was found between improvement following laser therapy and baseline parameters, except for the location of PWS.

## Introduction

A port-wine stain (PWS) is a congenital capillary abnormality with an incidence of 0.3–0.5%. PWSs are often pink in color with a flat onset and become hypertrophic and darker with age. Although the lesion primarily affects the head and neck, it may extend to the trunk and limbs and lead to physical and psychological suffering. Difficulty in interpersonal connections and a variety of mental issues have been described in such cases [1, 2].

Pulsed dye laser (PDL) therapy is now the gold standard management for PWS [3]. PWSs can be refractory and so resistant to standard PDL treatment in a proportion of individuals. The physical aspect of PWS can substantially impact these people [4].

The need for new treatments for resistant port-wine stains has prompted the development of new remedies. Using topical rapamycin (sirolimus) in conjunction with pulse dye therapy has been one of the innovative therapeutics for PWS. Many studies have yielded good outcomes for this combination, with improvement up to 50% even with resistance to PDL treatment [5, 6]. However, other trials have found no significant difference in clinical outcomes between treatment by PDL alone or with topical sirolimus [7, 8].

The 577-nm yellow laser has been judged suitable for vascular lesions due to its capacity to exclusively target oxyhemoglobin. Its added benefit over the copper bromide laser (formed of 90% yellow light and 10% green light) is that it lowers the hyperpigmentation risk in darker-skinned individuals. Furthermore, unlike PDL, it does not need a costly dye kit or a cooling like intense pulse light (IPL) [9]. Our study aimed to evaluate the prognostic variables influencing port-wine stain treatment with a 577-nm yellow laser.

## Patients and methods

This prospective cohort study involved 42 patients with PWS diagnosed based on clinical assessment and underwent laser treatment from June 2020 to May 2022. The Local Ethical committee approved the study, and written informed consent was obtained.

Patients with photosensitivity, keloid or hypertrophic scar tendency, or fibrosis were excluded.

All patients were treated using 577-nm yellow laser sessions (QuadroStarPRO YELLOW^®^ Asclepion Laser Technologies, Germany) at 4-week intervals for 6–10 sessions (laser treatments were terminated when the PWS decreased by 80%). Using the scanner handpiece (1 mm spot size, 80% coverage), the sessions began with a fluence dosage of 17 J/cm^2^. Every session, the mean dosage was raised by 2 J/cm^2^ to a maximum dose of 23 J/cm^2^ according to the tolerance of the patients. The pulse duration ranged between 20 and 32 ms. After the laser, a cold application with ice was administered for 30 min. All participants were instructed to use sunblock daily.

Before and four weeks after laser therapy, photos of the lesions were taken. Two dermatologists independently assessed the findings. Treatment success was considered an improvement of 80% or more in lesion color or size. Degree of improvement was classified as marked (≥ 75%), moderate (50% to < 75%), mild (25% - <50%) and poor (< 25%).

The data was analyzed using Statistical Package for Social Science (SPSS for Windows, Version 21.0. Armonk, NY: IBM Corp.). The mean and standard deviation (mean ± SD) were used to describe the parametric numerical data. The frequency and percentage were used to describe the non-numerical data. The ANOVA and Chi-square test were used to compare different degrees of improvement. P-value < 0.05 was considered significant.

## Results

This study involved 42 patients (31 females and 11 males) with PWS. The mean age of the patients was 23.1 ± 7.3 years, 29 patients (69%) were of skin type IV, and 13 (31%) were of type V. According to the PWS color, 27 patients (64.3%) were presented with red color, and 15 (35.7%) with pink color. The lesion in the mid-cheek was present in 20 (40%) patients, 4 (8%) patients were in the lips, 3 (6%) patients in the lateral cheek, 23 (46%) patients in the forehead, 13 (26%) patients in the chin, 8 (16%) patients in the upper lip, 9 (18%) of patients had PWS in the lower lip, and one patient had PWS in the neck (Table 1).

**Table 1:** Clinical data of studied patients

	Cases (*n* = 42)
Age, mean ± SD	23.12 ± 7.33
Gender, n (%)	
Males	11 (26.2)
Females	31 (73.8)
Skin phototype, n (%)	
IV	29 (69)
V	13 (31)
Color of PWS, n (%)	
Red	27 (64.3)
Pink	15 (35.7)
Site of PWS, n (%)	
Forehead	4 (9.5)
Mid-cheek	16 (38.1)
Lateral cheek	6 (14.3)
Lips	11 (26.4)
Neck	1 (2.4)
Chin	4 (9.5)

**Fig. 1:**
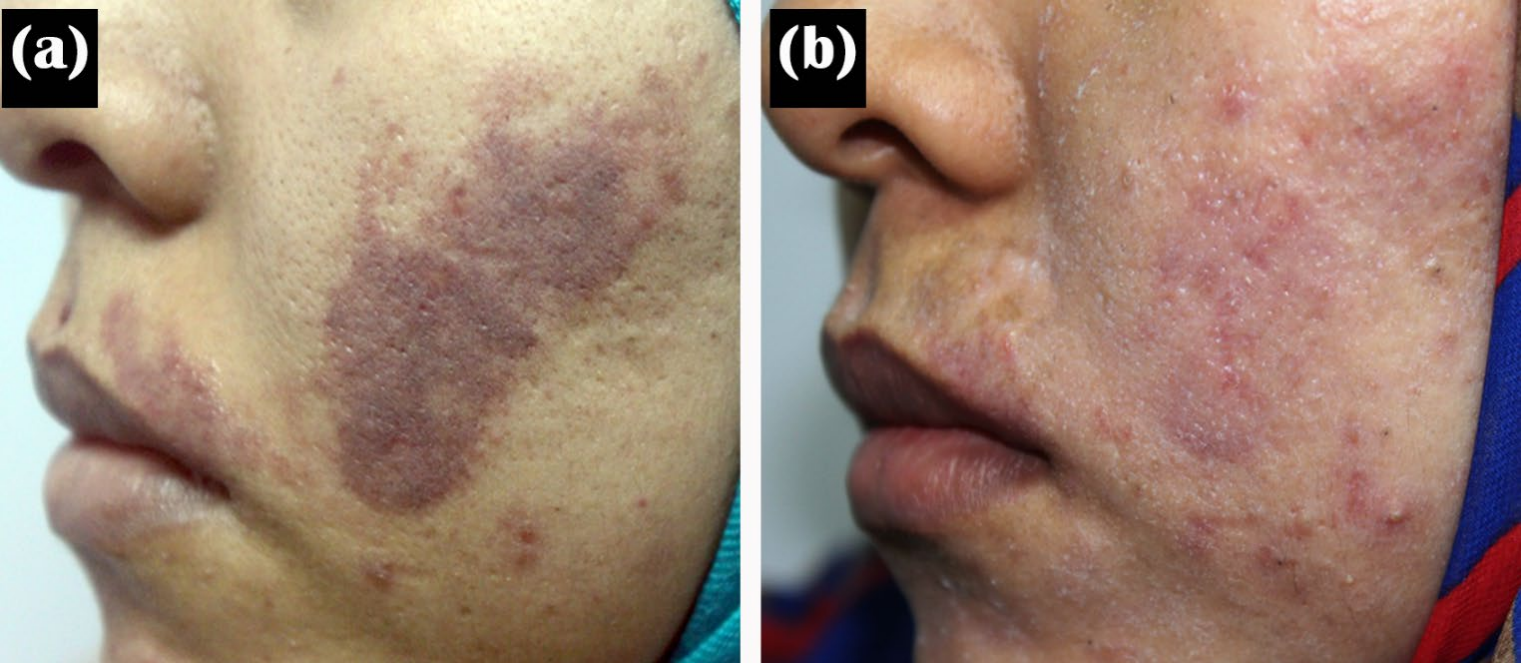
A 28-year-old female patient with PWS was treated by yellow laser (**a**) at baseline and (**b**) after eight treatment sessions

**Fig. 2:**
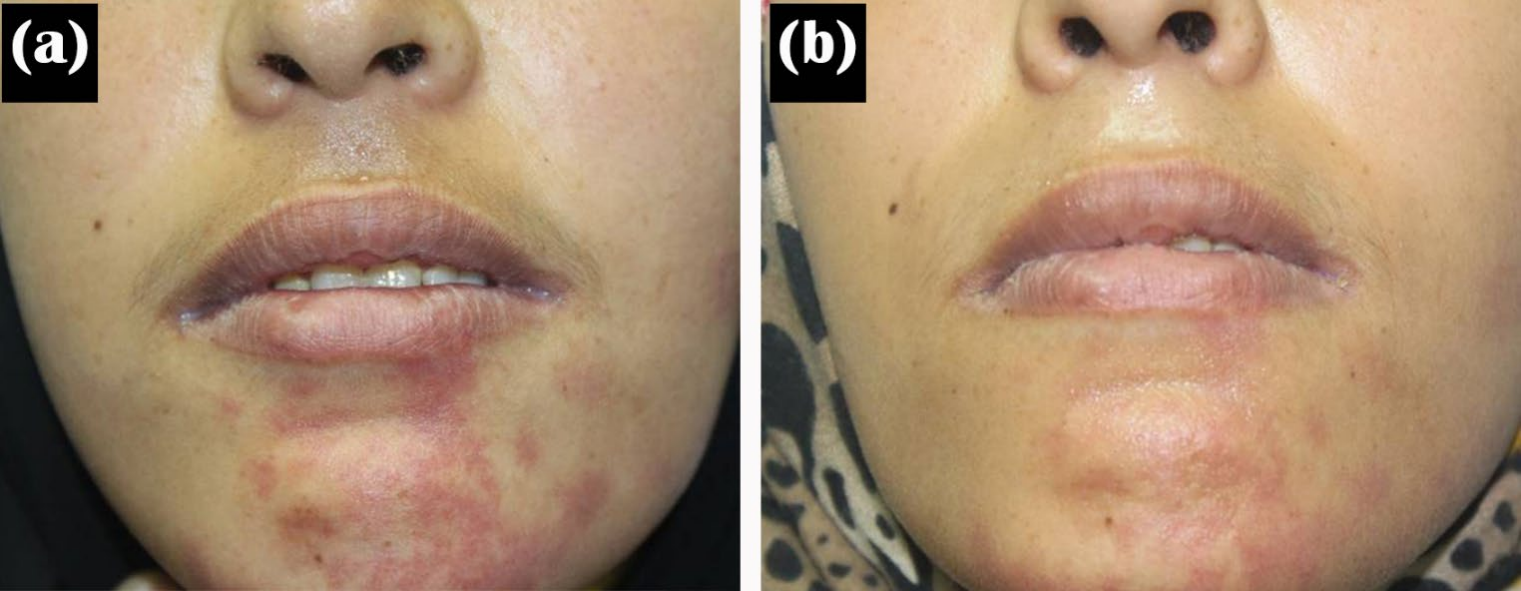
A 31-year-old female patient with PWS was treated by yellow laser (**a**) at baseline and (**b**) after ten treatment sessions

After treatment, the mean percentage of improvement was 54.64 ± 23.84. There was marked improvement in 7 (16.7%) patients, moderate improvement in 17 (40.5%) patients, mild improvement in 10 (23.8), and poor improvement in 8 (19%) patients (Figs. 1 and 2).

There was a statistically significant association between improvement and the gender of the patients and the site of the lesion (*p* = 0.028, *p* = 0.001, respectively). However, there was no significant correlation between the degree of improvement and the age of the patients or the skin type or color of PWS (*p* > 0.05 for each) (Table 2).

**Table 2:** Correlation between different degrees of PWS improvement and other parameters

	Marked	Moderate	Slight	Poor	*p*-value
**Age**	26.0 ± 8.85	22.06 ± 7.86	22.10 ± 3.63	24.13 ± 8.7	0.635
**Gender**,** n (%)**					
Females (*n* = 31)	5 (71.4)	13 (76.5)	10 (100)	3 (37.5)	0.028*
Males (*n* = 11)	2 (28.6)	4 (23.5)	0 (0)	5 (62.5)	
**Skin phototype**,** n (%)**					
IV (*n* = 29)	3 (42.9)	11 (64.7)	8 (80)	7 (87.5)	
V (*n* = 13)	4 (57.1)	6 (35.3)	2 (20)	1 (12.5)	0.237
**Color of PWS**,** n (%)**					
Red (*n* = 27)	5 (71.4)	4 (23.5)	4 (40)	2 (25)	0.14
Pink (*n* = 15)	2 (28.6)	13 (76.5)	6 (60)	6 (75)	
**Site**,** n (%)**					
Forehead (*n* = 4)	0 (0)	1 (5.9)	3 (30)	0 (0)	0.001*
Mid-cheek (*n* = 16)	4 (57.1)	2 (11.8)	7 (70)	3 (37.5)	
Lateral cheek (*n* = 6)	2 (28.6)	4 (23.5)	0 (0)	0 (0)	
Lips (*n* = 9)	1 (14.3)	6 (35.3)	0 (0)	4 (50)	
Neck (*n* = 1)	0 (0)	0 (0)	0 (0)	1 (12.5)	
Chin (*n* = 4)	0 (0)	4 (23.5)	0 (0)	0 (0)	

*p-value was significant

A linear regression analysis was performed to examine the contributions of different parameters as predictors in explaining the variance in improvement scores in the studied patients. The results showed that the site of PWS can significantly predict the improvement (*p* < 0.001). At the same time, other baseline characteristics, including age, sex, phototype, and color, were not associated and cannot act as predictors for improvement (Table 3).

**Table 3:** Multiple linear regression analysis for factor predicting degree of improvement in the studied patients

	Unstandardized coefficients		Standardized coefficients	Regression t-test	*p*-value
	B	SE	Beta		
**(Constant)**	-7.963-	41.439		-0.192-	0.849
**Age**	0.174	0.519	0.054	0.336	0.739
**Site**	-14.054-	10.461	-2.168-	-5.835-	< 0.001*
**Skin type**	13.769	8.478	0.270	1.624	0.113
**Color**	-1.747-	8.917	-0.036-	-0.196-	0.846
**Sex**	4.893	10.200	0.091	0.480	0.634

*p-value was significant; B: Regression coefficient; SE: Standard error

## Discussion

Despite advances in PWS therapeutic approaches, managing laser-resistant PWS, either due to lesion heterogeneity and/or limits of the used laser system, is still tricky. Most available therapeutic interventions have a short therapeutic window and a greater prevalence of side effects [10]. Earlier research investigated various treatment options for resistance to PWS lesions [11].

The yellow laser generates 100% yellow light at 577-nm, the perfect wavelength for vascular disorders [12]. Copper bromide lasers were used before emitting 90% yellow and 10% green light. Furthermore, the green wavelength was blamed for the adverse effects and poor success rate since it caused post-inflammatory hyperpigmentation. Because the yellow laser emits solely yellow light, it is an excellent choice for treating vascular disorders. The primary benefits of PWS therapy with a yellow laser are that it may be used effectively in dark-skinned people and has a minimal risk of scarring and erythema [13].

Prior research on the efficacy of 577/585-nm PDL in managing PWS found that full clearance happened in 25% of cases, 70% showed 50% or more lightening, and 20-30% responded poorly [14]. Unfortunately, few trials have evaluated the effectiveness of the 577-nm yellow laser in managing PWS. In our study, 7 (16.7%) patients improved markedly, 17 (40.5%) moderately improved, 10 (23.8%) mildly improved, and 8 (19%) patients poorly improved.

Mohamed et al. [15] employed the yellow laser to treat facial vascular disorders and found that a single pass of the 577-nm yellow laser resulted in a substantial improvement (> 50%) in PWS in around 64.5% of cases and bad outcome noticed in 16.2% of cases.

Several factors influence the effectiveness of laser therapy. A greater effect can be obtained at a younger age, with a purple lesion and with superficial and smaller vasculature [16]. Poor effects in specific individuals may be attributable to the dynamic nature of the vascular chromophore and variations in blood vessel size, depth, and intimal thickness [17]. While some reports have shown that lasers are more effective in treating PWS at a younger age, this topic is still debatable [18–20]. In addition, laser therapy for PWS may pose greater challenges for individuals with darker skin due to elevated melanin levels, heightening the chances of adverse effects like hyperpigmentation and scarring [21]. An analysis of 241 patients with Fitzpatrick skin types IV-VI revealed that adults who underwent PDL treatment displayed a wider spectrum of outcomes, ranging from enhancements in appearance to hyperpigmentation, hypopigmentation, and scarring. The most successful outcomes were typically observed in infants with small PWS located over bony facial areas, such as the central forehead [22]. Patients born with port wine stains should undergo early laser treatment for the best outcomes. Delay in treatment may worsen in nodularity over time and compromise the outcome [23].

Conversely, Mohamed et al. [16] found no significant link between improvement following 577-nm yellow laser therapy and patient ages, sex, duration of PWS, skin phototypes, or location of lesions.

Our study showed a significant correlation between improvement with gender and the site of PWS. However, there is no significant correlation between the degree of improvement with age, skin photo type or color of PWS. Given that skin phototypes IV and V are prevalent in our country, this study primarily addresses these types. Consequently, the results may not be generalizable to populations with a different distribution of skin types.

While other baseline characteristics, such as age, sex, phototype, and color, were not associated and could not predict improvement, linear regression analysis demonstrated that the lesion site could predict improvement, with PWS at cheek and lips showing a more significant improvement than other sites. In the future, by increasing laser-induced thrombosis and complete luminal blockage of PWS vasculature, site-specific yellow laser therapy may be a potential therapeutic choice for targeting therapy-resistant blood vessels.

When selecting a laser for managing PWSs, it is essential to consider cost and design. The yellow laser is practical, lightweight, and it does not need dye, gel, or cooling. Kapicioglu et al. [12] said this laser system was successful and dependable in treating face erythema, erythematotelangiectatic rosacea, and facial telangiectasia.

Except for a few individuals with mild to severe erythema that faded in 12–24 h following treatment with the 577-nm yellow laser, no side effects were detected [17]. Also, in the current study, the only adverse effects mentioned by the patients were irritation and temporary erythema. However, this variance can be tolerated, occurs during the first sessions, particularly at high fluence, and diminishes over future sessions.

This study uniquely assesses the prognostic factors affecting the PWS treatment by 577-nm yellow laser. However, it has several limitations, including the short follow-up period, the little variation of age and skin phototypes, and the lack of an assessment of various therapies and histological findings.

## Conclusion

The yellow laser is a successful therapy choice for PWS; however, multiple therapy sessions are necessary for deep-seated lesions, and some lesions do not resolve entirely. Also, there was no significant link between improvement following 577-nm yellow laser therapy and baseline parameters, except for the location of lesions.

## Data Availability

No datasets were generated or analysed during the current study.
